# Development of a dual-chamber derivatization method for the determination of cyanide in sodium nitroprusside and its preparation via HS-GC-ECD

**DOI:** 10.1016/j.jpha.2025.101353

**Published:** 2025-06-02

**Authors:** Jinqi Zheng, Xinyu Zhao, Caixia Li, Chenxiao Yan, Pingping Chen, Xiao Gu, Liya Hong, Su Zeng

**Affiliations:** a College of Pharmaceutical Sciences, Zhejiang University, Hangzhou, 310058, China; b Zhejiang Institute for Food and Drug Control, Hangzhou, 310052, China; c Green Pharmaceutical Collaborative Innovation Center of Yangtze River Delta Region and College of Pharmaceutical Science, Zhejiang University of Technology, Hangzhou, 310014, China

**Keywords:** Cyanide, Headspace gas chromatography, Dual-chamber, Sodium nitroprusside

## Abstract

The acute toxicity of cyanide and its pharmaceutical residues has fueled interest in the development of analytical methods for its determination, particularly for sodium nitroprusside (SNP), a widely used vasodilator with potential cyanide residues. In this study, a dual-chamber derivatization bottle was designed to establish an interconnected gas environment, thereby facilitating chloramine T-mediated cyanide conversion to cyanogen chloride (CNCl) without direct contact with SNP. Subsequent determination of the analytes was undertaken using a headspace-gas chromatography-electron capture detector (HS-GC-ECD). The challenges of analyzing cyanide and the rapid degradation of SNP were addressed simultaneously. The method was subjected to rigorous validation, encompassing specificity, linearity, limit of detection (LOD), limit of quantitation (LOQ), accuracy, precision, and robustness. The validation process revealed a notable degree of linearity within the range of 0.012–1.56 μg/mL, with a LOQ of 12.0 ng/mL. The method was found to be both accurate and precise, thus satisfying the requisite criteria. This method facilitates reliable cyanide monitoring in degradation-prone pharmaceuticals.

## Highlights

• An HS-GC-ECD method was developed to quantify cyanide in sodium nitroprusside.
• The method exhibited linearity, specificity, precision, and low LOD and LOQ values.
• The dual-chamber device was designed to achieve dual-chamber derivatization.
• Method is suitable for quality control analysis.

## Graphical abstract

**Figure:**
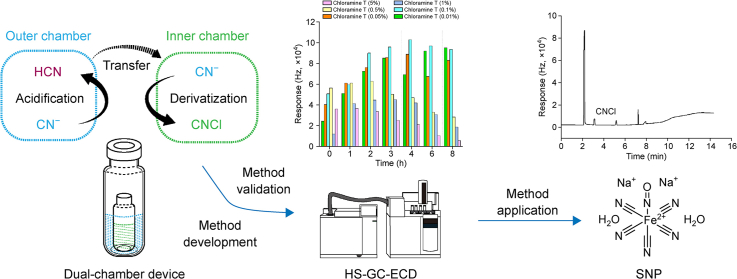

## 1 Introduction

Cyanide poisoning continues to represent a critical global public health concern. Once absorbed into the bloodstream, cyanide ions (CN^−^) exhibit a strong affinity for the Fe^3+^ ions present in cytochrome *c* oxidase. This interaction stabilizes a non-functional enzyme complex that effectively disrupts electron transport, thereby inhibiting cellular respiration [1]. The extant literature indicates that cyanide is an extremely toxic substance, with an acute toxic dose of 20 μg/kg and a lethal dose ranging from 0.5 to 3.5 mg/kg [2], Consequently, advanced chemical regulations, including Classification, Labelling and Packaging (CLP) [3], the Toxic Substances Control Act (TSCA) [4], and the Globally Harmonized System of Classification and Labelling of Chemicals (GHS) [5], classify cyanide as a highly toxic substance that requires strict control.

Sodium nitroprusside (SNP), a short-acting venous vasodilator, has been employed since the 1920s for the treatment of hypertensive crises and acute heart failure [6]. The therapeutic action of SNP primarily stems from the release of nitric oxide (NO), the inhibition of myosin complex enzymes, and the induction of vasodilation [7]. Following intravenous administration, SNP undergoes metabolic degradation to release CN^−^, most of which reacts with thiosulfate ions to yield thiocyanate, which has low toxicity. Another biological mechanism involves competitive binding to Fe^3+^ in cyclooxygenase (COX), causing severe toxicity. Furthermore, the unstable SNP is susceptible to photolytic and pH-dependent decomposition, resulting in the generating toxic CN^−^ as byproducts [8]. This poses significant risk to patient safety. The cyanide limit of SNP for injection in this study was set at 8 μg/g according to the Chinese Pharmacopoeia [9]. For the raw SNP material, a stricter cyanide limit of 5 μg/g was applied. Given that the injection formulation is not stable enough, the limit of SNP for injection was adjusted to 15 μg/g.

The extant analytical methodologies for cyanide detection principally comprise high performance liquid chromatography (HPLC) [10], gas chromatography (GC) [11], ion chromatography (IC) [12], flow injection [13], HPLC-mass spectrometry (HPLC-MS) [14], and GC-MS [15]. Akiyama et al. [10] employed an HPLC-fluorescence detector (FLD) for post-column derivatization detection of cyanide in bean paste, achieving a limit of quantitation (LOQ) of 0.1 μg/mL. Bruin et al. [11] utilized combination of GC and a nitrogen-phosphorus detector (NPD) for the detection of cyanide in plasma, achieving an LOQ of 0.1 μg/mL. However, this method was not suitable for trace detection. The IC is widely regarded as the gold standard for direct cyanide detection due to its superior sensitivity and specificity. Ghosh et al.’s [12] IC method achieved an LOQ of 1.45 ng/mL in polysaccharide vaccines, however, the electrode surface requires frequent cleaning. Campanella et al.’s [15] GC-MS method with pentafluorobenzene bromide alkylation achieved a LOQ of 17 ng/mL, but at the cost of prolonged workflows.

**Fig. 1:**
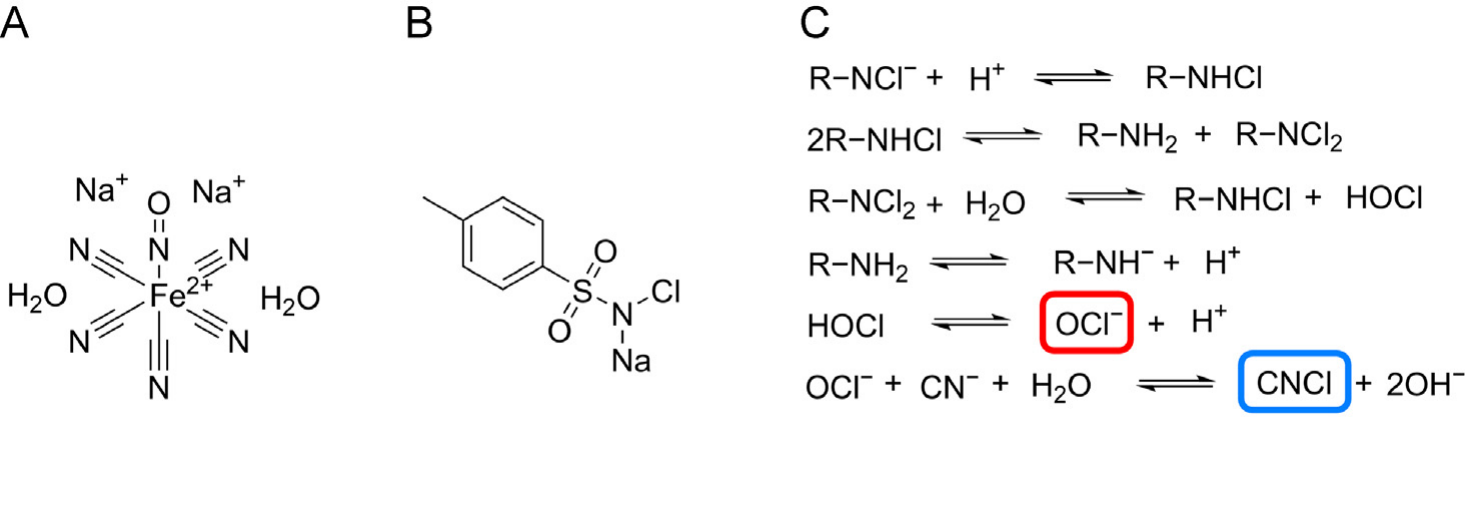
The structures of sodium nitroprusside (SNP) and chloramine-T and the schemes of derivatization reaction. (A, B) The structures of SNP (A) and chloramine T (B). (C) The process of active chlorine (OCl^−^ in the red frame and cyanogen chloride (CNCl) in the blue frame) generated in chloramine T solution (R = CH_3_–C_6_H_4_–SO_2_).

The detection of cyanide in SNP poses analytical challenges due to the instability of SNP and the chemical properties of cyanide. The direct derivatization strategy [14] is not suitable for overcoming the instability of SNP. The strategy [15] for dual-chamber systems requires offline processing of the derivatives, which hinders real-time analysis. It is essential to establish an online system for simultaneous cyanide derivatization and analysis. Chloramine T, a common derivatization reagent for cyanide detection, generates active chlorine which converts cyanide to cyanogen chloride (CNCl) via the pathway shown in Fig. 1. This process produces OCl^−^ and R–NH^−^ (R=CH_3_–C_6_H_4_–SO_2_), where Cl^+^ from OCl^−^ reacts with CN^−^ to form CNCl. Headspace-GC (HS-GC) combined with highly sensitive detectors, such as electron capture detector (ECD), is widely used for the analysis of volatile trace toxicants and is suitable for our study.

In this study, an HS-GC-ECD method was developed for the detection of residual cyanide in SNP formulations. A key innovation is a dual-chamber system that avoids direct contact of SNP with chloramine T. This design prevents SNP degradation while converting cyanide to CNCl for online HS-GC-ECD analysis. The approach improves both the analytical sensitivity and the operational safety.

## 2 Materials and methods

### 2.1 Materials

Five batches of SNP injection (strength, 2 mL:50 mg) and SNP raw material were obtained from Hainan Puli Co., Ltd. (Haikou, China), four batches of SNP for injection (strength, 50 mg) were obtained from Wuhan Renfu Co., Ltd. (Wuhan, China), Yuekang Co., Ltd. (Beijing, China), Jincheng Haisi Co., Ltd. (Jincheng, China), and Dandong Yichuang Co., Ltd. (Dandong, China), and potassium cyanide (KCN) (purity ≥ 98.0%) standards and chloramine T were purchased from Sinopharm Co., Ltd. (Beijing, China). Phosphoric acid was purchased from TEDIA (Fairfield, OH, USA). The 5% glucose mixture was purchased from Dalian Otsuka Co., Ltd. (Dalian, China). Pure water was prepared using a Milli-Q system (MilliporeSigma, Burlington, MA, USA).

### 2.2 Instrument conditions

Method development and validation were performed on an Agilent 7890B GC (Agilent Technologies, Santa Clara, CA, USA) and 7697A HS combined with an Agilent DB-WAXetr capillary column (30 m × 0.25 mm, 0.5 μm film thickness). A METTLER MS 205DU analytical balance (Mettler-Toledo, Zurich, Switzerland) was used for sample preparation. Pure water was prepared using a Milli-Q system (MilliporeSigma). The dual-chamber device was consisted of a 20 mL brown HS vial and a 2 mL screw vial (Agilent Technologies).

The optimized HS oven conditions were set at 50 °C with 20 min of equilibration. The sample loop and transfer line were operated at 90 and 100 °C, respectively. Nitrogen at 1.0 mL/min was used as the carrier gas. The injection port temperature was maintained at 200 °C, in split mode at a 10:1 ratio with an injection volume of 1.0 mL. The ECD was operated at 300 °C with nitrogen at 30 mL/min, and the column temperature was 50 °C for 5 min with a ramp of 30 °C/min to 200 °C for 4 min, resulting in a total run time of 14.3 min.

### 2.3 Solution preparation

#### 2.3.1 Derivatization solution preparation

An appropriate amount of chloramine T was dissolved and diluted with pure water, resulting in a final concentration of 0.1 mg/mL derivatization solution.

#### 2.3.2 Standard solution preparation

A 1% phosphoric acid aqueous solution was utilized as a blank solution. 100 mg of KCN was dissolved and diluted into a 100 mL volumetric flask with pure water, resulting in a mother liquor with a KCN concentration of 1 mg/mL. The standard stock solution was then diluted from the mother liquor with 0.1 M sodium hydroxide (NaOH), and a CN^−^ concentration of 50 μg/mL was obtained. Standard solutions (limit concentration) were then gradually diluted from the standard stock solutions with 0.1 M NaOH solution to obtain a final CN^−^ concentration of 0.125 μg/mL (SNP raw material), 0.2 μg/mL (SNP for injection), and 0.375 μg/mL (SNP injection). Subsequently, a series of working standard solutions were prepared by dilution from the standard solution with 0.1 M NaOH, yielding concentrations of 0.012, 0.036, 0.072, 0.181, 0.362, 1.04, and 1.56 μg/mL of CN^−^.

#### 2.3.3 Sample solution preparation

Appropriate amounts of SNP raw material, SNP for injection, and SNP injection were taken and then dissolved and diluted with pure water to obtain sample solutions for different formulations; their SNP concentrations were all 25 mg/mL.

Appropriate amounts of SNP raw material, SNP for injection, and SNP injection were dissolved and diluted with pure water, and then an appropriate amount of standard stock solution was added, resulting in spiked sample solutions for three CN^−^ limit concentrations (75%, 100%, and 125%) and three formulations. The CN^−^ limit concentration was equivalent to that of standard solutions of different formulations, and the concentrations of SNP in spiked sample solutions were all 25 mg/mL.

### 2.4 Dual-chamber derivatization

**Fig. 2:**
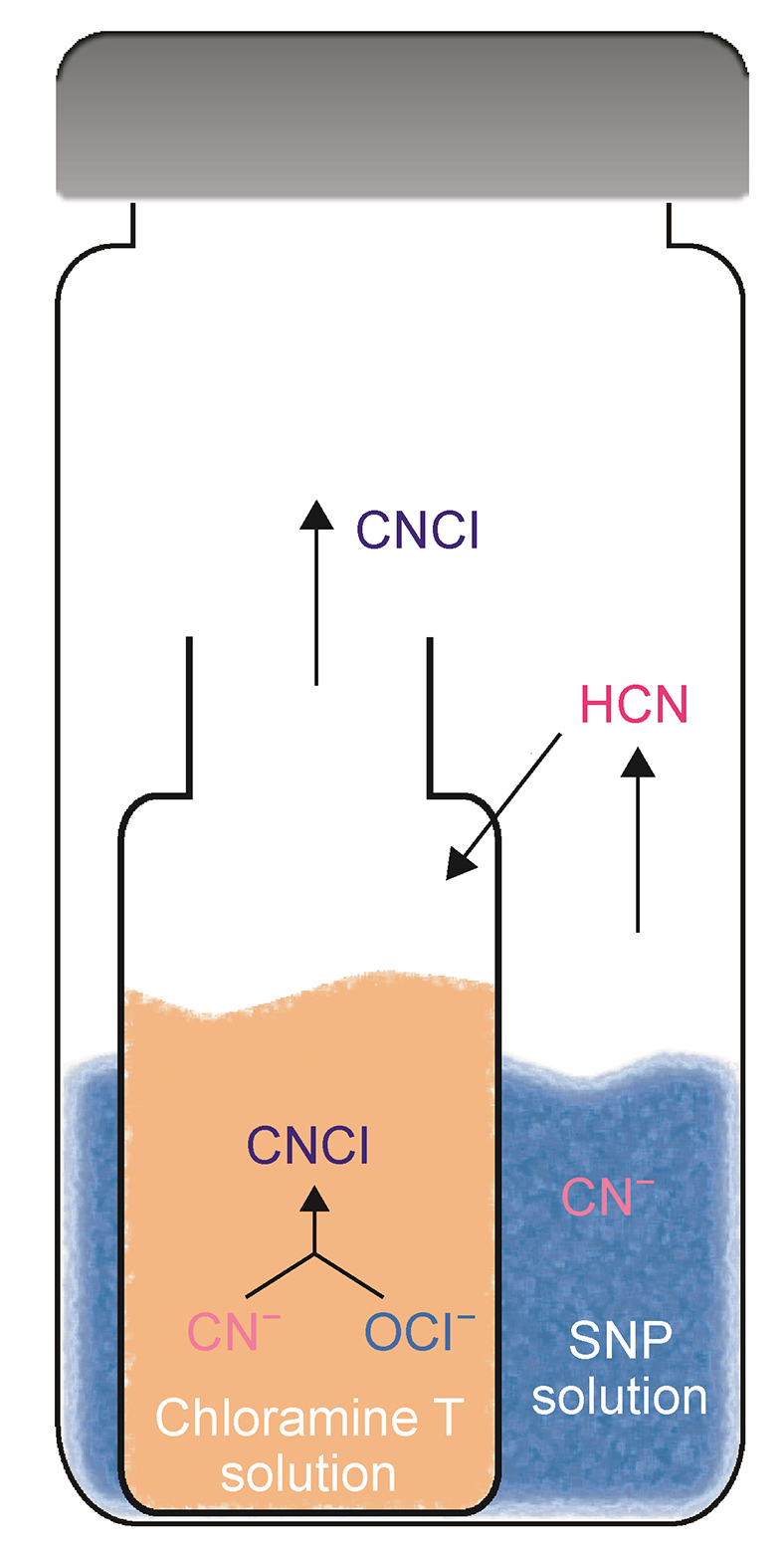
The formation of developed dual-chamber device and reaction mechanism within it. CNCl: cyanogen chloride; HCN: hydrogen cyanide; CN^−^: cyanide ion; SNP: sodium nitroprusside.

The custom dual-chamber derivatization bottle features a nested design, and a 2 mL inner screw vial (derivatization solution, CNCl generation chamber) was placed within a 20 mL outer HS vial (sample solution, hydrogen cyanide (HCN) generation chamber) with a cap, as shown in Fig. 2. Initially, 1.0 mL of sample mixture was introduced into the outer bottle, followed by the addition of 10 μL of phosphoric acid. The inner vial containing 0.5 mL of chloramine T solution was then immediately placed inside the outer vial, and the device was then sealed tightly. Finally, the sealed outer bottle was placed in an oven, heated at 50 °C for 1.5 h and transferred to HS-GC-ECD for detection.

## 3 Results and discussion

### 3.1 Method development

**Fig. 3:**
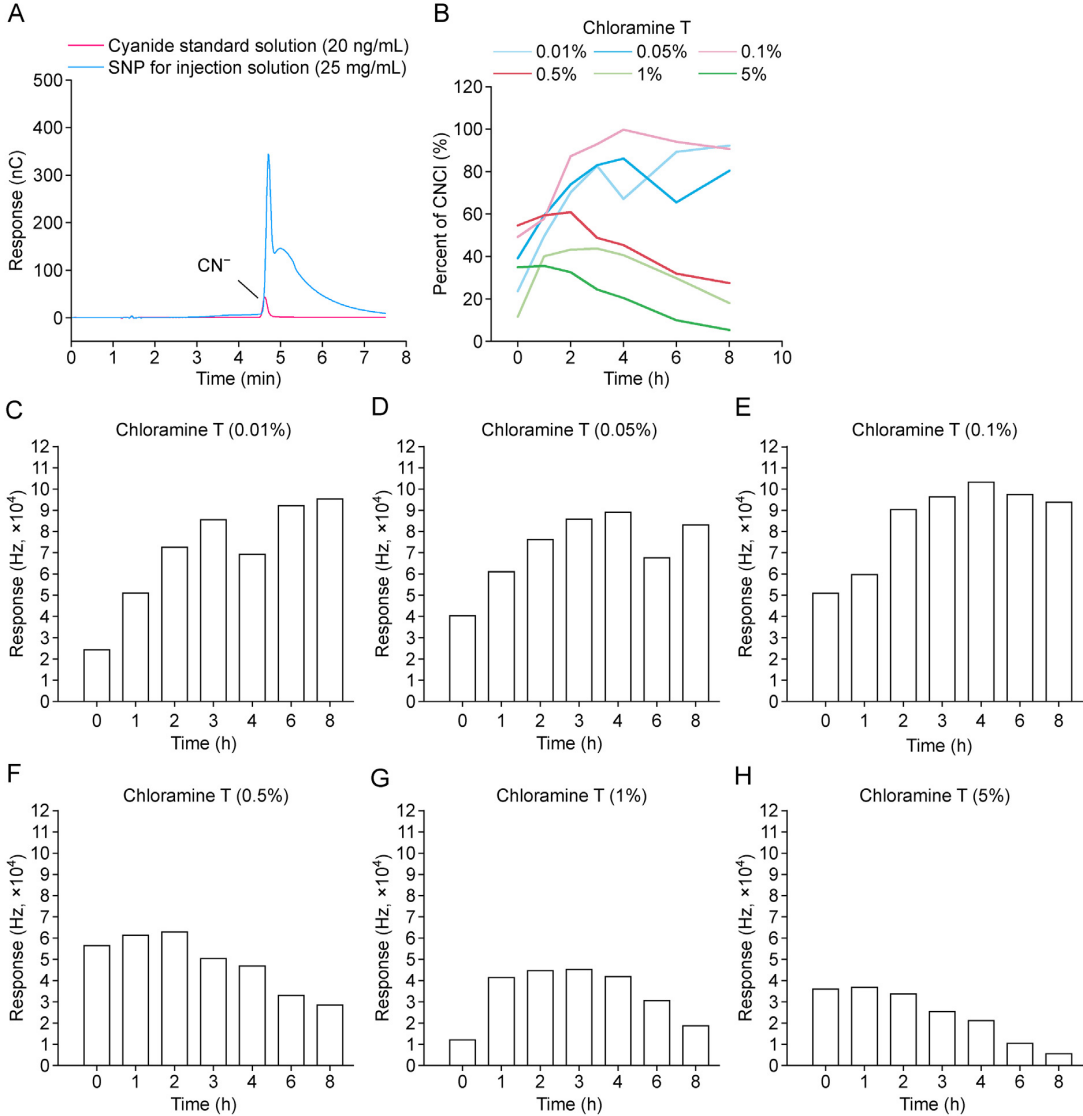
The results of ion chromatography (IC) experiment and derivatization reagent concentration optimization. (A) The chromatogram for 20 ng/mL cyanide standard solution and 25 mg/mL sodium nitroprusside (SNP) for injection using IC in method development. (B) The percent of cyanogen chloride (CNCl) response derived from different concentrations of chloramine T with different room temperature placement time. (C–H) The changes in CNCl content with the increase of the standing time at room temperature under the influence of different chloramine T concentration: 0.01% (C), 0.05% (D), 0.1% (E), 0.5% (F), 1% (G), and 5% (H).

As demonstrated in the preceding studies, the direct detection of cyanide by IC has been reported on multiple occasions. Its application to SNP analysis with NaOH eluent resulted in a coeluting cyanide peak, as shown in Fig. 3A, which indicated that alkaline-mediated SNP degradation generated spurious cyanide signals. This observation was consisted with the findings in previous literature [8]. Adjusting the eluent to neutral or slightly acidic conditions proved to be inadequate for the detection requirements for CN^−^.

Direct detection proved inadequate for the quantification of residual cyanide in SNP, thus necessitating the adoption of derivatization detection in this study. It has been demonstrated in several studies that the utilization of chloramine T to convert cyanide into CNCl [13] can enhance the retention of cyanide. To achieve the requisite sensitivity, GC-ECD was employed for the detection of electronegative analytes, particularly halogenated compounds such as CNCl. The volatility of CNCl was capitalized on by adopting HS-GC-ECD to detect residual cyanide in SNP.

### 3.2 Optimization of the derivatization method

During preliminary experiments, the direct derivatization HS-GC-ECD method involved immediate mixing of the sample mixture with chloramine T, but exhibited a similar effect to IC. The hypothesis was thus formulated that the slightly alkaline aqueous chloramine T solution (pH 9–10) would induce SNP degradation, generating cyanide and producing false-positive results. This observation confirmed the previous conclusion based on IC that SNP was unstable under alkaline conditions. We proposed that the chloramine T solution generated active chlorine with oxidizability, triggering structural instability of SNP.

Consequently, direct derivatization between chloramine T and the sample solution was not a viable option. Dual-chamber-based derivatization strategies for cyanide analysis have been reported in the extant literature [15]. The sample solution in a chamber was acidified to convert free CN^−^ into HCN, which was then transferred under hermetic conditions to another chamber for gas-phase trapping. The collected HCN was then derivatized with derivatization solution. This approach effectively avoids matrix interference from complex substrates (soil or food matrices), thereby preventing direct contact between the derivatization reagent and the sample. However, this conventional dual-chamber method has several critical limitations. The alkaline trapping solution necessitated post-collection processing, introducing the risks of exogenous contamination, which could compromise analytical accuracy and potential cyanide leakage, which could pose safety hazards. Consequently, the volatile CNCl employed in this study was not compatible with the offline transfer of this dual-chamber system.

The present study proposes a novel device that has been designed to combine combined with HS-GC-ECD, thus circumventing the requirement for offline transfer of either the trapping solution or derivatization products.

### 3.3 Optimization of derivatization conditions

#### 3.3.1 Solvent optimization

In order to guarantee the stability of SNP and the generation of HCN, the sample solutions must remain acidic. An investigation was conducted into various acidifying reagents utilized in the solution. Hydrochloric acid and nitric acid were excluded from further consideration due to their high volatility, which could interfere with GC analysis and potentially damage the liner and capillary column. Consequently, we examined the effects of sulfuric acid and phosphoric acid on the derivatization reaction. Based on the designed dual-chamber derivatization device, the sample solution was treated with 0.1%–10% sulfuric acid or phosphoric acid in conjunction with 0.1% chloramine T. Both acids were effective for derivatization, confirming that CN^−^ can be effectively converted to HCN under acidic conditions. The choice of 1% phosphoric acid was ultimately determined by its safety and convenience.

#### 3.3.2 Degradation of the derivatization product

Chloramine T, a prevalent derivatization reagent, generates active chlorine within aqueous solutions. When chloramine T was used to derive CN^−^, it triggered rapid CNCl formation with excellent GC compatibility. However, a notable problem with this method is the instability of CNCl during determination, which limited the reliability of the detection. The employment of a 1% chloramine T solution as the derivatization solution was found to result in a progressive decrease in the peak area of CNCl at room temperature, thereby hindering the accuracy of the determination. This finding indicated that post-formation degradation of CNCl resulted in a reduction in its content.

Eq. (1) $$\text{CNCl}+H_{2}O\;\to \;{\text{CNO}}^{-}+{\text{Cl}}^{-}+2H^{+}$$

Eq. (2) $$\text{CNCl}+{\text{OH}}^{-}\;\to \;{\text{CNO}}^{-}+{\text{Cl}}^{-}+H^{+}$$

According to Eqs. Eq. (1), Eq. (2)), CNCl in the chloramine T solution underwent slow hydrolysis via H_2_O and OH^−^ to produce CNO^−^ and Cl^−^. However, the reported hydrolysis rates are negligible [16], suggesting that the degradation of CNCl in this study was likely to have originated from non-hydrolytic pathways. In combination with the extant literature [17], the findings of the present study indicate that the degradation of CNCl was predominantly correlated with the active chlorine concentration, that is, the chloramine T level in the derivatization solution. It was necessary to optimize the process through mechanistic analysis of the established CNCl degradation pathway.

#### 3.3.3 Optimization of the derivatization reagent concentration

Eq. (3) $$\text{CNCl}+{\text{OCl}}^{-}\;\to \;\text{CNOCl}+{\text{Cl}}^{-}$$

The aqueous chloramine T solution exhibits a weak alkaline tendency (pH 9–10), which is essential for the absorption of atmospheric HCN and the subsequent generation of CN^−^ necessary for derivatization. The active chlorine, predominantly in the form of OCl^−^, generated in the aqueous chloramine T solution exerts a significant influence on both the rate of derivatization and the stability of CNCl [18]. The degradation of CNCl might proceed via Eq. (3). Consequently, the relationship between the chloramine T concentration and derivatization efficiency was further examined.

To optimize the chloramine T concentration for derivatization, a series of standard solutions were prepared with concentrations ranging from 0.01% to 5% of chloramine T, then subjected to an incubation process at room temperature for various time intervals (1–8 h). Following this incubation period, analysis was conducted using HS-GC-ECD analysis. The results are shown in Fig. 3B–H below. At a concentration of 0.1% chloramine T, the maximum level of CNCl can be observed after 2 h of derivatization, and it can be stable for a considerable time. Conversely, at concentrations greater than 0.1% chloramine T, a decline in CNCl was observed after it attained its maximum level. Chloramine T concentrations of 0.5%, 1%, and5 % have been shown to cause more than 50% degradation of CNCl within 8 h of preparation, demonstrating concentration-dependent relationship between chloramine T and CNCl degradation. At lower chloramine T concentrations (0.01% and 0.05%), CNCl exhibited enhanced stability albeit with a slower derivatization rate. The chloramine T concentration of 0.1% was therefore identified as the optimal choice, achieving a balanced compromise between derivatization efficiency and CNCl stability.

#### 3.3.4 Optimization of the derivatization temperature and time

**Fig. 4:**
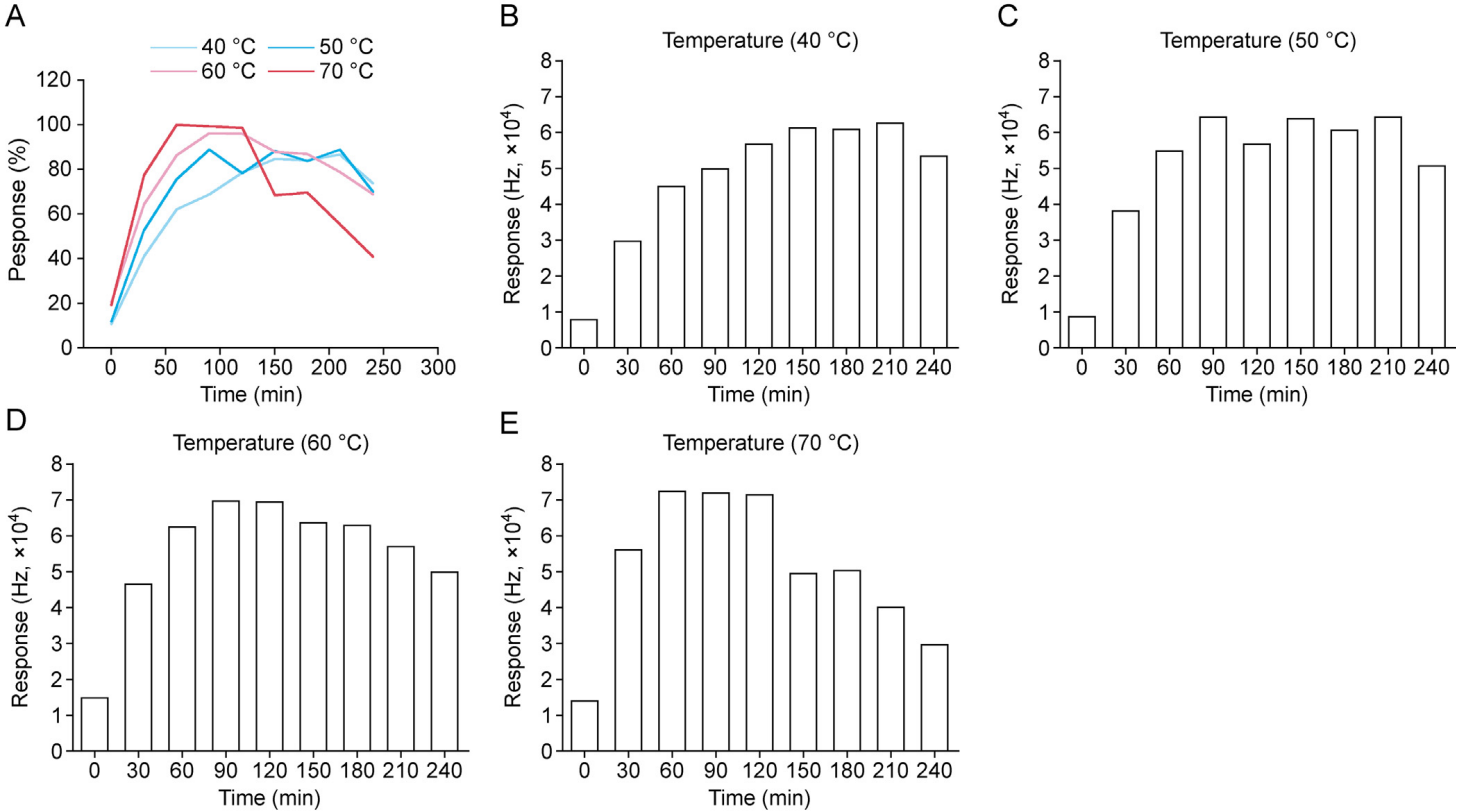
The results of derivatization temperature and derivatization time optimization. (A) The percent of cyanogen chloride (CNCl) response derived by 0.1% chloramine T in different derivatization temperature and time. (B–E) Response of CNCl at different derivatization temperature and time: derivatization temperature at 40 °C (B), 50 °C (C), 60 °C (D), and 70 °C (E).

The derivatization temperature and time are required to achieve the optimal conditions. Multiple sample solutions were prepared and incubated in an oven at temperatures ranging from 40 to 70 °C for 0–4 h, following the corresponding equilibrium temperature in HS oven. The results are shown in Fig. 4. The principal observation was that an elevated derivatization temperature accelerated equilibrium attainment and increased the CNCl content. Conversely, the concurrent acceleration of CNCl degradation at elevated temperature complicated the control of derivatization. Furthermore, an elevated HS oven temperature may lead to an unexpected chromatographic water peak and mass loss of the stationary phase liquid in the capillary column. It was determined that 50 °C derivatization temperature and 1.5 h derivatization time would provide optimal reproducibility and rapid completion.

The optimized HS-GC-ECD method was established as follows: the dual-chamber device was transferred to a HS autosampler after incubation, followed by equilibration at 50 °C for 20 min, and then injected into a GC-ECD equipped with a DB-WAXetr capillary column. Separation was achieved through a temperature-programmed process with a total runtime of 14.3 min. CNCl contains halogen atom, which means excessive concentrations of CNCl in GC system may cause adverse effects such as contamination of the capillary column and detector. To address this issue, an extended elution time has been implemented in the chromatographic method to ensure complete elution of CNCl and other chlorides, thereby maintaining column clean. Additionally, the ECD is maintained at elevated temperature throughout operation to prevent detector contamination.

### 3.4 Cyanide extraction and derivatization

This study addressed the limitations of cyanide extraction and derivatization through the implementation of a novel dual-chamber device. The residual cyanide predominantly existed as CN^−^ in the sample solution, and derivatization was initiated by acidification of the sample solution in the outer chamber with 10 μL of phosphoric acid. This was followed by immediate insertion of an inner chamber that had been preloaded with 0.1% chloramine T solution and subsequent sealing. This enabled acid-mediated conversion of CN^−^ to volatile HCN. The gaseous HCN diffused into the inner chamber, where it reacted with OCl^−^ to form volatile CNCl, thereby achieving selective cyanide extraction independent of SNP interference while eliminating potential matrix effects.

The gas-liquid partitioning of CNCl was promoted by incubation in the oven and HS sampler within the closed system, thus completing the sample pretreatment prior to HS-GC-ECD analysis. A key advantage of this integrated design is that it enabled the online execution of cyanide extraction, derivatization, and instrumental detection within a single device.

Prior to HS sampling, pressurization equilibration is a step that may induce leakage of analytes. To ensure consistent sealing integrity across parallel measurements, it is essential that all external chambers were securely capped using an automatic crimping device. The volume of sample solution in the outer chamber was maintained below 1.0 mL, and volumes in excess of this amount would increase the risk of sample solution migration into the inner chamber during operation, causing cross-contamination.

The inner chamber, which was prepared under ambient conditions, required meticulous cleaning when it contained chloramine-T. Residual chloramine-T adsorption on the inner chamber surface must be avoided to eliminate interference in trace-level quantification. Notably, the absence of organic solvents and structurally simple analytes in this experimental system serves to minimize the likelihood of contamination from extraneous sources.

### 3.5 Method validation

**Fig. 5:**
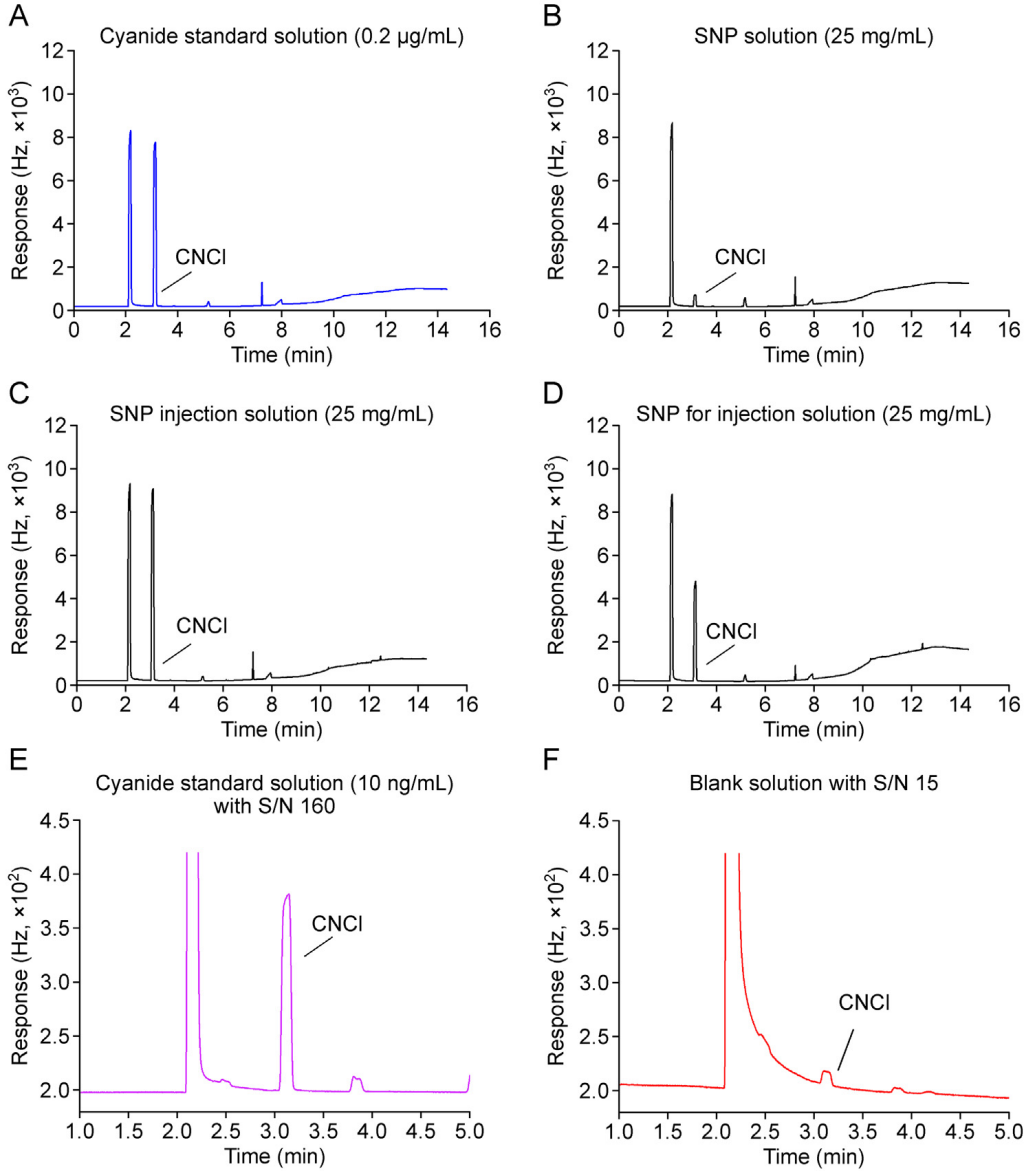
Typical chromatograms of standard solution, sample solutions, and blank solution: (A) 0.2 μg/mL cyanide standard solution; (B) 25 mg/mL sodium nitroprusside (SNP) solution; (C) 25 mg/mL SNP injection solution; (D) 25 mg/mL SNP for injection solution; (E) 10 ng/mL cyanide standard solution with signal to noise ratio (S/N) 160; and (F) blank solution with S/N 15. CNCl: cyanogen chloride.

The present method was thoroughly validated in accordance with the ICH guidelines for SNP raw material, SNP for injection, and SNP injection including specificity, linearity, limit of detection (LOD)/LOQ, accuracy, precision, specificity, robustness and stability. The typical chromatograms of 0.2 μg/mL CN^−^ standard solution, 25 mg/mL SNP, 25 mg/mL SNP for injection, and 25 mg/mL SNP injection are shown in Figs. 5A–D.

#### 3.5.1 Specificity

Post-derivatization analysis of both the blank solution and the sample solution revealed no interference with the detection process, thereby confirmed the high specificity of the developed method. The retention time of CNCl was 3.1 min. Notably, trace levels of cyanide were observed in the blank solution, despite employing muti-source water as the solvent. This confirmed persistent blank contamination at trace concentrations, which was consistent with a previous report [19].

#### 3.5.2 Linearity

A series of working standard solutions were diluted to CN^−^ concentrations of 0.012, 0.036, 0.072, 0.181, 0.362, 1.04, and 1.56 μg/mL for construction of the calibration curve. Linear fitting was performed using the least squares method to examine the relationship between the analytes concentration and chromatographic response. A calibration curve was subsequently established, achieving the equation of *Y* = 122610*X* – 950.56. The coefficient of determination (*R*^2^) value was 0.9993, thereby demonstrating excellent linearity within the CN^−^ concentration range of 0.012–1.56 μg/mL (equivalent to 0.48–62.40 μg/g).

#### 3.5.3 Sensitivity

LOQ solutions were analyzed to evaluate the sensitivity of developed method, as shown in Figs. 5E and F, and the signal to noise ratio (S/N) for the LOQ was determined to be approximately 10-fold greater than that of the blank solution, which effectively eliminated blank-induced signal interference while maintaining method validity. The LOQ for this method was determined to be 12.0 ± 1.3 ng/mL (mean ± standard deviation (SD)%, *n* = 6), indicating that the method developed in this study had sufficient sensitivity to assay CN^−^ concentration from SNP. In addition, the LOD was validated as 4.2 ng/mL and can be used to identify CN^−^ in SNPs.

#### 3.5.4 Accuracy and recovery

Spiked sample solutions for three CN^−^ limit concentrations (75%, 100%, and 125%) and three formulations were injected in order to analysis to evaluate the accuracy of the method. Following the subtraction of the background, the cyanide recovery rate and relative SD (RSD)% across all spike levels were calculated. The results are shown in Table 1. The average recoveries of SNP raw material, SNP injection, and SNP for injection were 98.7%–110.8%, 108.4%–111.3%, and 95.8%–106.5%, respectively, with the RSD% values all below 10%, thus confirming the accuracy of the developed method.

**Table 1:** Summary of the method accuracy and precision result.

Analytes	Accuracy (75% level)		Accuracy (100% level)		Accuracy (125% level)		Precision (100% level)	
	Recovery (%) (*n* = 3)	RSD (%)	Recovery (%) (*n* = 3)	RSD (%)	Recovery (%) (*n* = 3)	RSD (%)	Intra-day RSD (%) (*n* = 6)	Inter-day RSD (%) (*n* = 24)
SNP	108.3	3.2	98.7	9.5	110.8	7.2	3.9	10.0
SNP injection	108.4	3.9	109.9	6.0	111.3	2.1	8.5	7.0
SNP for injection	106.5	2.9	95.8	5.0	103.6	0.2	3.9	11.5

RSD: relative standard deviation; SNP: sodium nitroprusside.

#### 3.5.5 Precision

The spiked sample solutions of the three formulations were spiked at 100% CN^−^ limit concentrations and analyzed on a daily basis for four consecutive days (24 h interval). The RSD% values for one day and four consecutive days were calculated to evaluate the intra-day and inter-day precision of the method. The results are shown in Table 1. The RSD% values were all less than 15%, thus confirming that the method developed in this study exhibited excellent reproducibility and precision.

#### 3.5.6 Stability

The derivatization step employed in this study was time-sensitive to the time after preparation of the solution, which greatly affected the stability of the solution and accuracy of the experiment. The 100% limit spiked sample solutions of three dosage forms were prepared and stored at room temperature after 1, 2, 4, 8, and 12 h. Post-derivatization analysis (Table 2) confirmed that the CNCl generated in the three SNP formulation sample solutions remained stable at 12 h (the peak areas of the SNP raw material, SNP injection, and SNP for injection at 12 h were 93.2%, 91.6%, and 86.1% greater than those at 0 h, respectively). Therefore, utilizing conservative estimates, approximately 45 parallel samples could be efficiently processed and analyzed within a typical analytical sequence without encountering stability issues, thereby validating the practical applicability of the method.

**Table 2:** The validation results of the method stability and robustness.

Analytes	Stability (%)						Robustness (%)							
	0 h	1 h	4 h	8 h	12 h	Mean ± SD%	190 °C	200 °C	210 °C	Mean ± SD%	0.9 mL/min	1.0 mL/min	1.1 mL/min	Mean ± SD%
SNP	100.0	97.5	101.9	97.0	93.2	97.9 ± 3.3	108.2	100.0	111.0	106.4 ± 5.7	100.0	100.0	102.3	100.8 ± 1.3
SNP injection	100.0	101.7	97.7	101.5	91.6	98.5 ± 4.2	115.1	100.0	107.6	107.6 ± 7.6	107.9	100.0	91.3	99.7 ± 8.3
SNP for injection	100.0	100.5	104.2	96.9	86.1	97.5 ± 6.9	117.4	100.0	117.1	111.5 ± 10.0	107.8	100.0	96.6	101.5 ± 5.7

SNP: sodium nitroprusside; SD: standard deviation.

#### 3.5.7 Robustness

The robustness of the method was verified by adjusting the flow rate of the carrier gas and the injection port temperature for the injection of 100% spiked sample solutions of SNP and its preparation. After derivatization for 1.5 h at 50 °C, the samples were injected and analyzed, as shown in Table 2, under adjusted chromatographic parameters, the peak area of CNCl exhibited less than 20% deviation from the original chromatographic conditions, thereby confirming the robustness of the developed method.

### 3.6 Batch analysis

Three batches of sample solutions were subjected to post-derivatization analysis to determine residual cyanide. The results are presented in Table 3. Trace amounts of cyanide were detected in the three SNP formulations. The residual cyanides in the SNP raw material, SNP injection, and SNP for injection were 1–3 μg/g (<5 μg/g), 12–13 μg/g (<15 μg/g), and 2–6 μg/g (<8 μg/g), respectively, which were all effectively controlled within the study-established limits.

**Table 3:** The results of determination of cyanide in sodium nitroprusside (SNP), SNP injection, and SNP for injection.

Analytes	Lot #1 (μg/g)	Lot #2 (μg/g)	Lot #3 (μg/g)	Target limit (μg/g)
SNP	2	1	3	5
SNP injection	12	13	13	15
SNP for injection	6	2	6	8

### 3.7 Comparison with the reported methods

Compared with existing derivatization methods for cyanide determination, the developed method demonstrated superior sensitivity approaching that of MS. The cyanide extraction principle has been shown to be compatible with other dual-chamber systems, thereby permitting applications in diverse matrices including biological specimens, foodstuffs, and soil samples. Importantly, a notable finding of the study was the achievement of concurrent cyanide extraction and derivatization within the dual-chamber system, substantially reducing the duration of pretreatment within 2 h. The online derivatization injection protocol eliminated offline transfer of derivatized products, thereby increasing operational safety and convenience. However, potential limitations such as compromised reproducibility might exist under a dual-chamber system and HS injection, coupled with contamination risks from insufficient cleansing of the inner vial. A detailed comparative summary of different methods developed for CN^−^ in various matrices is presented in Table 4 [[10], [11], [12], [13], [14], [15]].

**Table 4:** Comparative summary of cyanide determination methods.

Method	Technique	Cyanide extraction; extraction time (h)	Matrix	Derivatization reagent; derivatization time (min)	Limit of quantitation (μg/mL)	Linearity (μg/mL)	Refs.
Direct method	GC-nitrogen phosphorus detector	Extraction; 0.25 h	Human plasma	NR; NR	1.0	1–100	[11]
	Ion chromatography	Dual-chamber system (offline); 0.8 h	Meningococcal conjugate samples	NR; NR	0.00145	0.00145–0.0931	[12]
Derivatization method	HPLC-FLD	Dual-chamber system (offline); 2 h	Sweetened bean paste	Chloramine T; NR	0.1	0.52–520	[10]
	Flow-injection	Distillation; NR	Water	Chloramine T; NR	2.0	2–100	[13]
	HPLC-MS	Centrifugation; 0.7 h	Swine plasma	Naphthalene-2,3-dicarboxaldehyde; NR	0.26	0.26–130	[14]
	GC-MS	Dual-chamber system (offline); 18.5 h	Soil	Pentafluorobenzyl bromide; 10 min	0.017	0.05–0.8	[15]

GC: gas chromatography; NR: not reported; HPLC: high performance liquid chromatography; FLD: fluorescence detector; MS: mass spectrometry.

## 4 Conclusions

As we known, it is a big challenge to detect cyanide in SNP with its instability and toxicity. An innovative isolation derivatization HS-GC-ECD method was developed to accurately quantify residual cyanide in SNP raw material, SNP for injection, and SNP injection in this study. The issue of poor retention, attributable to the high polarity of cyanide, was addressed through process of derivatization using chloramine T. Furthermore, an innovative dual-chamber device, consisting of an outer chamber and an inner chamber, was developed to resolve the structural instability of SNP, which hitherto precluded direct derivatization. Under a stabilized SNP structure, residual trace cyanide can be effectively derivatized without additional steps such as manual transfer, which not only minimizes the risk of transfer loss, but also improves the accuracy of the analysis. To further optimize the derivatization conditions, investigations were conducted into chloramine T concentration, derivatization temperature and time. The objective of these investigations was to optimize both the derivatization efficiency and the stability of CNCl.

In conclusion, we developed and validated a dual-chamber derivatization method for the determination of cyanide in SNP and its preparation via HS-GC-ECD in this study. This analytical method not only facilitates reliable cyanide monitoring in SNP, but also establishes a novel approach for cyanide detection in structurally unstable pharmaceuticals, effectively mitigating analytical interference from degradation of unstable API structures. In addition, further research is required to understand and improve the stability of CNCl.

## CRediT authorship contribution statement

**Jinqi Zheng:** Writing – original draft, Validation, Methodology, Formal analysis, Data curation. **Xinyu Zhao:** Writing – review & editing, Resources. **Caixia Li:** Resources, Project administration. **Chenxiao Yan:** Validation, Software. **Pingping Chen:** Visualization, Investigation. **Xiao Gu:** Supervision, Investigation. **Liya Hong:** Project administration. **Su Zeng:** Writing – review & editing, Supervision, Resources, Investigation, Data curation, Conceptualization.

## Declaration of competing interest

The authors declare that they have no known competing financial interests or personal relationships that could have appeared to influence the work reported in this paper.
